# *De novo* transcriptome assembly of shrimp *Palaemon serratus*

**DOI:** 10.1016/j.gdata.2016.12.009

**Published:** 2016-12-23

**Authors:** Alejandra Perina, Ana M González-Tizón, Iago F. Meilán, Andrés Martínez-Lage

**Affiliations:** a Department of Cell and Molecular Biology, Evolutionary Biology Group (GIBE), Universidade da Coruña, A Fraga 10, E-15008 A Coruña, Spain; b Centro de Investigaciones Científicas Avanzadas (CICA), Universidade da Coruña, 15071 A Coruña, Spain; c aCore, Falperra 36, E-15005 A Coruña, Spain

**Keywords:** RNA-seq, Illumina, *Palaemon serratus*, Transcriptome, Muscle, Larvae

## Abstract

The shrimp *Palaemon serratus* is a coastal decapod crustacean with a high commercial value. It is harvested for human consumption. In this study, we used Illumina sequencing technology (HiSeq 2000) to sequence, assemble and annotate the transcriptome of *P. serratus*. RNA was isolated from muscle of adults individuals and, from a pool of larvae. A total number of 4 cDNA libraries were constructed, using the TruSeq RNA Sample Preparation Kit v2. The raw data in this study was deposited in NCBI SRA database with study accession number of SRP090769. The obtained data were subjected to *de novo* transcriptome assembly using Trinity software, and coding regions were predicted by TransDecoder. We used Blastp and Sma3s to annotate the identified proteins. The transcriptome data could provide some insight into the understanding of genes involved in the larval development and metamorphosis.

**Specifications:**

**Table:** 

Organism/cell line/tissue	*Palaemon serratus*/muscle adults individuals and pool of larvae
Sex	N/A
Sequencer or array type	Illumina HiSeq2000
Data format	Raw or processed
Experimental factors	*De novo* transcriptome assembly of *Palaemon serratus*.
Experimental features	RNA was isolated from muscle of adults individuals and, from a pool of larvae. A total number of 4 cDNA libraries were constructed, using the TruSeq RNA Sample Preparation Kit v2. The obtained data were subjected to *de novo* transcriptome assembly using Trinity, and coding regions were predicted by TransDecoder. We used Blastp and Sma3s_v2 to annotate the identified proteins.
Consent	N/A
Sample source location	Artabro Gulf (43° 22′00″N, 8°28′00′′’W) in the northwest of Spain.

## 1 Introduction

The common littoral shrimp *Palaemon serratus* (Pennant, 1777) is a coastal decapod crustacean that inhabits the intertidal and subtidal soft-sediment of estuaries and rocky bottoms covered with seagrass and algae [1]. The world distribution covers the Atlantic Ocean, from Scotland and Denmark to Mauritania, and all the Mediterranean Sea, Marmara and the Black Sea [2]. The capture of *P. serratus* maintains a very important traditional activity in some fishing communities due to its high commercial value, mainly in North of Spain (up to 140€/kg on Christmas). In fact, the *P. serratus* fishery contributes annually more than ten million Euros to the European economy [3]. Despite its high economic value, the availability of genomic and transcriptomic data for this shrimp in public databases is limited. In addition to its ecological and commercial importance, these species have proved to be suitable indicator species in ecotoxicology [4], [5]. In this study, we performed *de novo* transcriptome assembly and annotation for *P. serratus* from adults individuals, and from a pool of larvae, by next-generation sequencing. These transcriptomic data provide useful information to reveal putative genes involved in the larval development and metamorphosis and help identify novel genes.

## 2 Experimental design, materials and methods

### 2.1 Animal materials

Specimens of *P. serratus* were collected from the Artabro Gulf (43° 22′00″N, 8°28′00′W) in the northwest of Spain. Animals were captured with a fish trap and some individuals were preserved in RNA*later*® (Life Technologies). The rest of them were carried alive to the laboratory where they were kept at 18 °C in an aerated aquarium and fed with frozen brine shrimp for at least 24 h, until larvae were released. All samples were kept at − 80 °C until they were processed.

### 2.2 RNA isolation, library construction and sequencing

RNA isolation and library construction was carried out at AllGenetics (A Coruña, Spain) according to the following procedure. RNA was isolated from muscle of adults individuals (Pser), and from a pool of larvae (LPser), using the reagent NZYol (NZYTech). Briefly, frozen samples were homogenised using a mortar and pestle under liquid nitrogen. 1 mL of NZYol was added directly to the homogenate, and transferred to a nuclease-free 1.5 mL tube. Then, we added 0.2 volumes of chloroform-isoamil alcohol (24:1), centrifuged the mixture, and recovered the supernatant into a new tube. One volume of ice-cold isopropanol was added, and the mixture was kept at − 20 °C overnight in order to precipitate the RNA. The samples were centrifuged, and the supernatant was discarded. The pellet was washed with 96% ethanol. The ethanol was discarded, and the pellet resuspended in a final volume of 30 μL. RNA concentration and integrity were measured in an Agilent 2100 Bioanalyzer. A total number of 4 cDNA libraries were constructed, using the TruSeq RNA Sample Preparation Kit v2 (Illumina Inc. San Diego, *CA*), strictly following the manufacturer's instructions. From each of the RNA samples, we constructed 2 different libraries (one ‘original’ library and its replicate). All the ‘original’ libraries were run in a HiSeq 2000 PE100 lane, whereas all the ‘replicates’ were run in a different HiSeq 2000 PE100 lane. Within each lane, the libraries were pooled in equimolar amounts, according to the quantication data provided by the Qubit dsDNA HS Assay Kit, before high throughput sequencing.

### 2.3 *De novo* transcriptome assembly, identification of protein coding region, and annotation

**Fig. 1:**
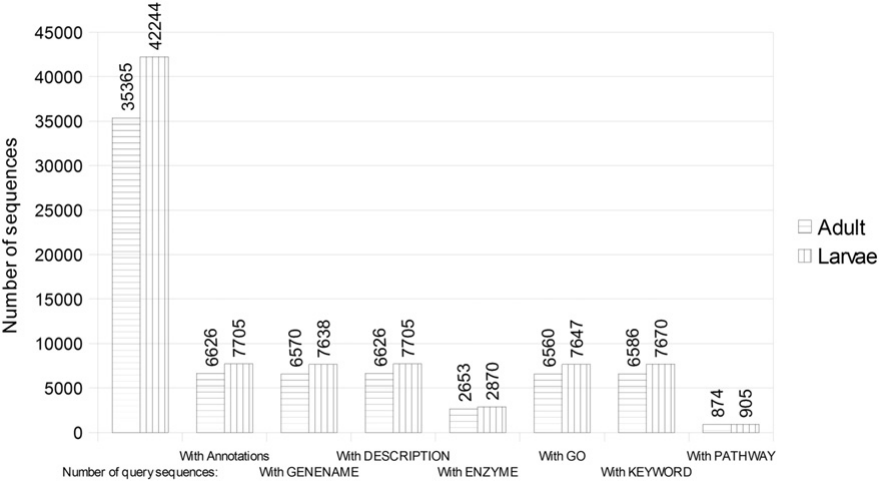
Comparison of the annotation of adult *vs* larvae transcriptome against Swiss-Prot database.

We obtained 9.5 and 7.5 GB of raw data from Pser and Pser_rep respectively (original and replicate respectively), and 11.6 and 7.7 GB of raw data from LPser and LPser_rep respectively, by paired-end sequencing (deposited in NCBI SRA database with study accession number of SRP090769). Quality control for the raw reads was performed using FastQC [6]. After the removal *Illumina* adaptors and filter sequences with the Trimmomatic v0.35 [7] a total of 65,765,083 cleaned reads were obtained from adults individuals of *P. serratus*, and 75,307,090 cleaned reads from larvae. The specific parameters to obtain high quality reads were: 1) cut the 12 bases from the start of the read, 2) trimming sequences by the end of them and based on the value of quality, establishing a minimum quality value 25 and, 3) removing reads with a length less than 40 nucleotides. These high quality reads were *de novo* assembled using Trinity software v.2.2.0 [8] with default parameters settings (K mer = 25). Detailed information on the *de novo* trasncriptome assembly is summmarized in Table 1. The coding regions prediction of assembled transcripts was carried out by TransDecoder (implemented in the Trinity software). The results showed 35,364 and 42,244 ORFs for adults and larvae, respectively. We carried out a local Blastp on the predicted proteins against NCBI non-redundant protein sequences (nr) database (September 2016) to predict the putative functions of the identified proteins. The Blastp results can be found in Supplementary material 1. The predicted proteins, too, were functionally annotated using a modified version of the Sma3s program [9], which allows the tracing of the source of each annotation and initially tries to discover the query sequences in the annotated database. It uses the UniProt database to assign gene names, descriptions and EC (Enzyme Commission) numbers to the query sequences and adds GO terms, UniProt keywords and pathways. The predicted amino acid sequences was used as input for two executions of the Sma3s, one against Swiss-Prot database (manually curated) and another against TrEMBL database (automatically annotated and not reviewed) from unannotated sequences against Swiss-Prot database. The annotation results and their statistics can be found in Supplementary material 2. An annotation statistic comparison of adult and larvae transcriptomes against Swiss-Prot database was summarized in Fig. 1. All large-scale computational analyses were performed on a high performance computing cluster, The Supercomputing Centre of Galicia (CESGA). The transcriptome data in this work will be usefully applied to study genes involved in the larval development and metamorphosis.

## Conflict of interest

The authors declare that they have no competing interests.

## Figures and Tables

**Table 1:** Summary of the *de novo* transcriptome assembly for *P. serratus*.

Index	Adults transcriptome	Larvae transcriptome
Total trinity ‘genes’	95,601	124,389
Total trinity transcripts	112,716	152,110
Percent GC	39.33	39.36
Contig N50	2311	2596
Median contig length	405	401
Average contig	996.97	1047.88
Total assembled bases	112,374,970	159,393,572
